# Comprehensive management application of sulodexide on proteinuria: 2 case reports

**DOI:** 10.3389/fmed.2025.1519604

**Published:** 2025-02-05

**Authors:** Li Zhou, Wenge Li

**Affiliations:** Department of Nephrology, Beijing China-Japan Friendship Hospital, Beijing, China

**Keywords:** proteinuria, chronic kidney disease, diabetic kidney disease, chronic diseases, sulodexide

## Abstract

Proteinuria is an important sign of kidney function decline and one of the common clinical manifestations in patients with chronic kidney disease and diabetic kidney disease, which are often accompanied by a gradual decline in kidney function. Therefore, effective management of proteinuria is of great significance for delaying the progression of kidney disease and protecting kidney function. Sulodexide is a mixture of fast-moving heparin and dermatan sulfate with anticoagulant, thrombolytic, anticardiovascular, lipid-lowering, and renal-protective effects. It has potential application value in the comprehensive management of proteinuria. This study reported two different chronic disease cases to explore the comprehensive management application of sulodexide in patients with long-term poor proteinuria control. Case 1 is a 45 years-old male with type 2 diabetes mellitus and hypertension accompanied by persistent proteinuria and intermittent edema in both legs. Case 2 is a 52 years-old female with chronic nephritis and rheumatoid arthritis accompanied by persistent proteinuria and edema in both legs. Two patients showed a decrease in urinary protein levels and stable control of the remaining signs of disease after the addition of sulodexide to their comprehensive treatment regimens, indicating that sulodexide plays an important role in the comprehensive management of proteinuria.

## Introduction

Proteinuria is one of the clinical manifestations of renal impairment, and its causes are diverse, involving immune system disorder, hypertension, diabetes, and other factors. These reasons lead to the damage of the renal filtration barrier, so that a large number of proteins cannot be recovered normally, and then leak out and deposit in various tissues and organs of the body (1). Proteinuria is a common clinical manifestation in patients with chronic kidney disease (CKD) and diabetic kidney disease (DKD). The presence of proteinuria indicates kidney dysfunction and is more likely to be a warning signal for the progression of kidney disease (2, 3). Effective management of proteinuria is of great significance for delaying the progression of kidney disease and protecting kidney function. Although existing treatment methods can relieve the symptoms of proteinuria to some extent, there are still challenges and limitations. Therefore, treatment and management strategies for proteinuria are the focus of research in the field of kidney disease.

Sulodexide is a compound preparation composed of fast-moving heparin and dermatan sulfate, which can prevent the degradation of heparan sulfate and restore the ion-selective permeability of the glomerular basement membrane to some extent. It shows potential application value in the comprehensive management of proteinuria (4, 5). Previous studies have shown that sulodexide has a significant effect on reducing the albumin excretion rate in diabetic patients (6). The long-term administration of low-dose sulodexide exhibits antiproteinuric and renal protective effects in patients with CKD caused by DN, primary glomerulonephritis, and hypertensive nephropathy (7). In these treatments, sulodexide has played an important role in protecting kidney function and delaying the progression of kidney disease to some extent.

This study reported the treatment of patients with chronic diseases who had long-term poor proteinuria control. We identified patients with an initial 24 h urinary protein level exceeding 3 g; one patient had diabetic kidney disease, while the other had non-diabetic kidney disease. These two patients had previously received multiple treatment regimens, but their proteinuria was not effectively controlled. To assess kidney function, we utilized a combination of clinical indicators including 24 h urinary protein, blood creatinine and estimated glomerular filtration rate (eGFR). We added sulodexide as a treatment strategy and closely followed up on its efficacy to explore the application of sulodexide in the comprehensive management of proteinuria.

## Case description

### Case 1

A 45 years-old male patient with a BMI of 29.07 kg/m^2^ came to the Department of Nephrology of our hospital in February 2020 due to “severe proteinuria and intermittent edema in both legs for 3 months.” Previous medical history: type 2 diabetes mellitus for 20 years, previous poor glycemic control, hypertension for 5 years, proteinuria for 17 years, urinary protein 13.6 g in December 2019, no kidney biopsy. The decision to forego a biopsy was based on the patient’s clinical presentation, medical history, and the available laboratory tests, which provided sufficient information to guide our management plan. This patient was diagnosed with type 2 diabetes mellitus, hypertension, hyperlipidemia, chronic renal failure, renal anemia, proteinuria, hyperkalemia, and arteriosclerosis obliterans.

The patient received comprehensive treatment in our hospital, including antihypertensive, hypoglycemic, lipid-lowering, and antiproteinuric therapies. Treatment regimen: the patient regularly takes olmesartan medoxomil tablets 20 mg once daily, olmesartan medoxomil hydrochlorothiazide tablets 20 mg once daily, nifedipine controlled release tablets 30 mg once daily, and arotinolol hydrochloride tablets 10 mg twice daily for blood pressure control; acarbose 100 mg three times daily, linagliptin 5 mg once daily, repaglinide 1 mg three times daily, and liraglutide injection for regular glycemic control; atorvastatin calcium tablets 20 mg once daily for blood lipid control; beraprost sodium tablets three times daily for comprehensive proteinuria control. In addition, there were records of taking roxadustat capsules 50 mg three times a week, half a bag of sodium zirconium cyclosilicate once daily, febuxostat tablets 80 mg once daily, mecobalamin tablets, silybin meglumine tablets, calcitriol soft capsules, bailing capsules, and okra capsules. During the comprehensive treatment, blood glucose was stable, anemia was well-controlled, blood potassium was controllable, creatinine in kidney function was stable and gradually decreasing, total blood protein was within the normal range, and only proteinuria was poorly controlled. Therefore, the patient’s proteinuria control regimen was adjusted: 2–4 sulodexide capsules (50–100 mg) were added twice daily (since 17 December 2021), beraprost sodium tablets three times daily, and finerenone tablets 10 mg once daily (since 7 December 2022). After taking sulodexide, the level of proteinuria decreased and was stably controlled.

**FIGURE 1:**
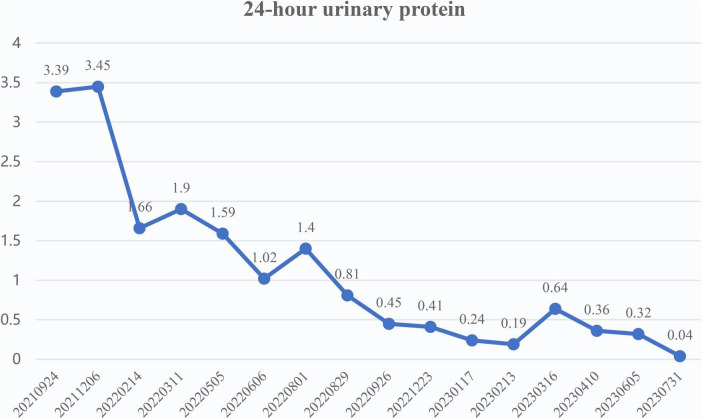
Trend of 24 h urinary protein (g/24 h) of case 1.

Follow-up observation of patients: from 24 September 2021 to 31 July 2023, follow-up observation was conducted on changes in hemoglobin, 24 h urinary protein, electrolyte, kidney function, blood glucose and other indicators. The total blood protein fluctuated between 64 and 72.8 g/L (normal range: 60–80 g/L) during the 2 years follow-up period; after taking sulodexide (17 December 2021), the urinary protein level was under control, the 24 h urinary protein kept decreasing, and the 24 h urinary protein decreased from 3.45 to 0.04 g/L during the follow-up period, as shown in Figure 1 (normal range: ≦ 0.15 g/24 h); the rest of the indicators: fasting blood glucose was maintained at 4.35–5.39 mmol/L, and blood glucose was stable; creatinine level fluctuated at 197.3–221.5 μmol/L (normal range: 53–106 μmol/L), and re-examination in July 2023 revealed a stable blood creatinine level of 220.6 μmol/L; moreover, the blood potassium and sodium fluctuated in the normal range fluctuation (Table 1). Treatment outcome: after the addition of sulodexide for comprehensive treatment in December 2021, the patient’s 24 h urinary protein continued to decline, and by July 2023, it decreased to 0.04 g/L. The creatinine was stable, blood potassium was controllable, blood glucose was stable, and the overall clinical symptoms were stably controlled.

**TABLE 1:** Changes in blood glucose, creatinine, protein and electrolytes in case 1.

Date	Fasting blood glucose (mmol/L)	Total protein quantification TP (g/L)	Creatinine (enzymatic method) CR (μ mol/L)	eGFR (ml/min)	24 h urinary protein (g/24 h)	K+ (mmol/L)	Na+ (mmol/L)
2022.12	5.39	69.4	204	33.06	0.41	4.9	139.8
2023.1	4.35	64.2	200.2	33.82	0.24	4.8	142.8
2023.2	5.78	66.9	197.3	34.42	0.19	5.6	140.4
2023.6	5.48	66.5	221.5	26.96	0.32	4.8	140.8
2023.7	4.64	70.2	220.6	32.01	0.04	4.5	141.4

### Case 2

**FIGURE 2:**
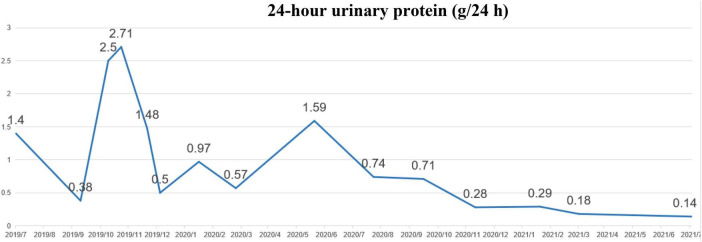
Changes in 24 h urinary protein between July 2019 and July 2021 in case 2.

**FIGURE 3:**
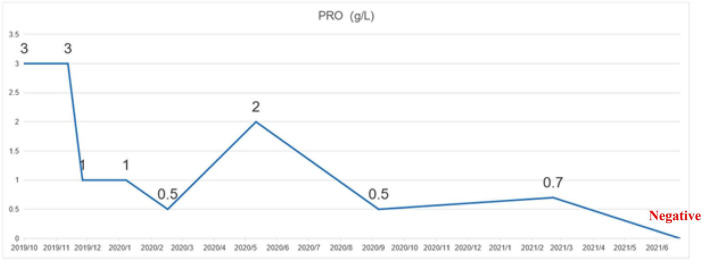
Change in urinary protein between October 2019 and June 2021 in case 2.

A 52 years-old female patient. Proteinuria 2–3 (+) was found for 2 years with more serious edema in both legs for 1 month, which had been treated with ARB + ciclosporin + decoction in other hospitals with the poor effect of comprehensive treatment. The patient received treatment at our hospital in July 2019, no kidney biopsy. Previous medical history: hypertension for 4 years, rheumatoid arthritis for 5 years. Physical examination: blood pressure 132/75 mmHg, with obvious edema in both legs; urine routine: PRO: 3 g/L; 24 h urinary protein: 1.66 g/24 h; blood biochemistry: TP: 62.4 g/L, ALB: 35.3 g/L, CHO: 6.77 mmol/L, LDL-C: 4 mmol/L; UA: 261 μmol/L, CR: 39.3 μmol/L. Diagnosis: chronic nephritis, rheumatoid arthritis, hypertension, hyperlipidemia, arteriosclerosis obliterans, and hepatic function abnormal. Patient medication: comprehensive treatment, including antihypertensive, lipid-lowering, rheumatoid arthritis immunosuppressive and antiproteinuric therapies. On 17 January 2020, the proteinuria control regimen was adjusted: 2 capsules/time (50 mg) of sulodexide were added twice daily. Following up to July 2021, the patient’s 24 h urinary protein returned to a normal level of 0.14 g/24 h, and urinary protein turned negative (Figures 2, 3).

## Discussion

Sulodexide is a highly purified mixture of glycosaminoglycans composed of 80% fast-moving heparin and 20% dermatan sulfate. As an antithrombin, sulodexide exhibits protective effects on the kidney and distant organs. In an animal model, the levels of serum urea nitrogen, creatinine, and inflammatory factors in sulodexide-intervened rats showed significant reductions. Furthermore, sulodexide can protect the kidney from histological changes and inhibit intraparenchymal apoptosis (8). In a mouse model of lipopolysaccharide-induced glomerulonephritis, sulodexide has a potential protective effect in experimental glomerulonephritis (9). In experimental studies, sulodexide, as a heterogeneous group of sulfated glycosaminoglycans, can prevent diabetic kidney morphological and functional changes, suppress the production of renal inflammatory cytokines and vascular growth factors, and improve endothelial dysfunction and albuminuria (10, 11). The DiNAS clinical trial was published in 2002 (6), which included 223 patients with type 1 and type 2 diabetes mellitus who had microalbuminuria or macroalbuminuria in 13 centers. After 4 months of treatment, the sulodexide group had a significant effect in reducing the albumin excretion rate in patients with type 1 and type 2 diabetes mellitus compared to the placebo group. The DAVET study was published in 2010 (12) and included 216 patients with type 1 and type 2 diabetes mellitus in 31 centers. After 3 and 6 months of sulodexide treatment, the albumin excretion rate in diabetic patients was significantly reduced, indicating that long-term low-dose administration of sulodexide in diabetic patients can effectively reduce the albumin excretion rate. A 6 months treatment with sulodexide 150 mg daily did not achieve 50% reduction of urinary protein excretion in Immunoglobulin nephropathy patients, but showed a tendency to increase the time-dependent anti-proteinuric effect (13). A meta-analysis published in 2015 included 10 studies on DN (14), of which eight studies were used to assess the efficacy of sulodexide on albumin excretion rates. The results showed that the albumin excretion rate in patients with DN was significantly reduced after the use of sulodexide. In a case-control study published in 2020, sulodexide 200 mg/day for 12 months was effective in maintaining proteinuria in patients with type 2 diabetes and non-nephrotic proteinuria but provided no added benefit in renal disease progression (15). Another meta-analysis published in 2021 included 34 studies related to DKD (16) and selected studies in which sulodexide was compared with placebo for analysis. The results showed that sulodexide significantly reduced the albumin excretion rate in patients with DKD. Among these treatment regimens, sulodexide can provide significant benefits to patients with kidney disease.

In this report, the criteria for selecting the typical cases focused on patients with CDS who had poor control of long-term proteinuria. In addition to the cases presented, we did encounter other patients who did not meet our selection criteria or exhibited different outcomes. For instance, some patients had milder degrees of proteinuria or had achieved better control of their proteinuria through medical interventions. These patients were not included in our study as they did not meet the criteria. The patient in case 1 had type 2 diabetes mellitus for more than 20 years, with previous poor glycemic control and a 5 years history of hypertension. Proteinuria appeared 17 years ago without obvious reasons, which were not typical at that time. Then, the urine protein increased without any special discomfort, and the symptoms of edema in both legs appeared in the past 3 months. From October 2007 to October 2011, the patient visited the superior hospital many times, but the diagnosis and treatment showed no improvement. In December 2019, the patient’s urinary protein level reached 13.6 g. After a regular regimen at our hospital, including antihypertensive, hypoglycemic, lipid-lowering, and comprehensive control of urinary protein (sulodexide + beraprost sodium tablets + finerenone tablets), the urinary protein was controlled. Besides, the patient underwent an ophthalmological examination and was diagnosed with retinopathy before treatment was initiated. Following the administration of sulodexide and subsequent monitoring, the patient’s retinopathy had resolved. The patient in case 2 had chronic nephritis and rheumatoid arthritis. Proteinuria (2+)–(3+) appeared 2 years ago without obvious reasons, with more serious edema in both legs for 1 month. She had been treated with a comprehensive treatment of ARB + ciclosporin + decoction in other hospitals (treatment cycle of about 2 years), but the effect was not good. The patient started taking sulodexide 2 capsules/time on 17 January 2020 in our hospital. After treatment, the patient’s 24 h urinary protein returned to a normal level of 0.14 g/24 h in July 2021, and the urinary protein turned negative. We assessed the stability of disease control through the gradual decrease in urinary protein and stabilization of kidney function with blood creatinine. The gradual reduction in urinary protein levels indicated an improvement in kidney function, whereas the stability of creatinine levels suggested that there was no significant deterioration in kidney function over time. These data provided evidence of effective disease control in the cases we reported.

This clinical report involves two patients with chronic diseases who had poor control of urinary protein during past symptomatic treatment and suffered from proteinuria for a long period. This persistent proteinuria not only affects the quality of life of patients but may also aggravate renal impairment. The addition of approximately 100 mg/day of sulodexide to the comprehensive treatment regimen of these two patients resulted in a significant change, with a decrease in the level of proteinuria and stable control of the remaining signs of disease. The doses of sulodexide used for managing proteinuria fell within the median range compared to previous studies, which administered 50–200 mg/day (7, 12, 13, 15). In summary, sulodexide, as an endothelial-protective drug, has a significant application effect on long-term proteinuria control when used in combination with other drugs. This finding provides a new treatment strategy for patients with chronic diseases such as CKD and DKD, which is expected to better control proteinuria and reduce renal impairment. As a kind of heparinoids, sulodexide has some anticoagulant effects, and its efficacy in preventing deep vein thrombosis has been fully demonstrated in previous studies. It is worth noting that such drugs often have the potential risk of inducing hemorrhage, whereas the risk of hemorrhage with sulodexide is relatively low. A study by Andreozzi et al. (17) confirmed that prolonged sulodexide administration has a comparable risk of hemorrhage to that of placebo in a population that has experienced unprovoked venous thromboembolism (VTE). The two patients involved in this clinical report, both whom had been taking sulodexide for more than 1 year. We tested coagulation, monitored liver function, and observed no signs of drug-induced allergic reactions or liver damage. Furthermore, we kept track of any unusual symptoms reported by the patients. No hemorrhage or other adverse reactions related to sulodexide were observed during the treatment, which also confirmed the safety of the drug in the clinical application of the CKD population. Although our cases have been followed up for a duration of one and a half years, we acknowledge that the current follow-up period may still be considered insufficient for a definitive conclusion on long-term effects. The clinical benefit of sulodexide in the treatment of proteinuria still requires further clinical verification of its universality and long-term effects, but this discovery undoubtedly provides a new idea for the comprehensive management of proteinuria.

This report is the first case study on the comprehensive treatment of proteinuria with sulodexide in patients with chronic diseases and long-term poor proteinuria control. This study had some limitations that should be considered when interpreting the findings. Firstly, the small sample size of patients limits the generalizability of the results to the broader population with kidney disease. Secondly, the lack of a control group further restricts the possibility to draw definitive conclusions regarding the efficacy and safety of sulodexide in comparison to other treatment modalities. Thirdly, the differences in patients with different types and severity of kidney disease may impact their comparability. Lastly, the case report, which focuses on certain medical metrics, does not comprehensively capture other factors such as infections, accidents, diarrhea, quality of life, and cardiovascular risks, which may also influence the potential long-term outcomes for patients. Future studies with larger cohorts, controlled designs, and extended follow-up periods are necessary to validate these findings and to better understand the role of sulodexide in managing proteinuria.

In conclusion, we reported two cases to explore the application of sulodexide in the management of patients with long-term poorly controlled proteinuria. One case involved diabetic kidney disease, the other involved non-diabetic kidney disease. After adding sulodexide to their comprehensive treatment plans, both patients achieved favorable long-term outcomes, with well-controlled proteinuria and stable serum creatinine levels. This report is a case study that provides preliminary evidence for the management of proteinuria with sulodexide. We will continue to explore the application of sulodexide in proteinuria management through further studies and provide more valuable treatment strategies for CKD clinical practice.

## Data Availability

The original contributions presented in this study are included in this article/supplementary material, further inquiries can be directed to the corresponding author.
